# Supplementary material for: Disentangling microbial networks across pelagic zones in the tropical and subtropical global ocean
Source: Nat Commun. 2024 Jan 2;15:126. doi: 10.1038/s41467-023-44550-y (PMC10762198; doi:10.1038/s41467-023-44550-y)
Supplement: Supplementary file 1 — Supplementary Information [file 41467_2023_44550_MOESM1_ESM.pdf]

Supplementary Information

Disentangling microbial networks across pelagic zones in the tropical and subtropical global ocean

Ina M. Deutschmann[1*], Erwan Delage[2,3], Caterina R. Giner[1], Marta Sebastián[1], Julie Poulain[4], Javier Arístegui[5], Carlos M. Duarte[6], Silvia G. Acinas[1], Ramon Massana[1], Josep M. Gasol[1], Damien Eveillard[2,3], Samuel Chaffron[2,3] and Ramiro Logares[1*]

[1]Institute of Marine Sciences (ICM), CSIC, Barcelona, Spain.

[2]Nantes Université, CNRS UMR 6004, LS2N, F-44000, Nantes, France.

[3]Research Federation for the study of Global Ocean Systems Ecology and Evolution, FR2022 / Tara Oceans GOSEE, Paris, France.

[4]Génomique Métabolique, Genoscope, Institut François Jacob, CEA, CNRS, Univ Evry, Université Paris-Saclay, Evry, France.

[5]Instituto de Oceanografía y Cambio Global, IOCAG, Universidad de Las Palmas de Gran Canaria, ULPGC, Gran Canaria, Spain.

[6] King Abdullah University of Science and Technology (KAUST), Red Sea Research Center (RSRC), Thuwal, Saudi Arabia.

[*]Corresponding authors: Ina Maria Deutschmann (ina.m.deutschmann@gmail.com) and Ramiro Logares (ramiro.logares@icm.csic.es)

Supplementary Figures

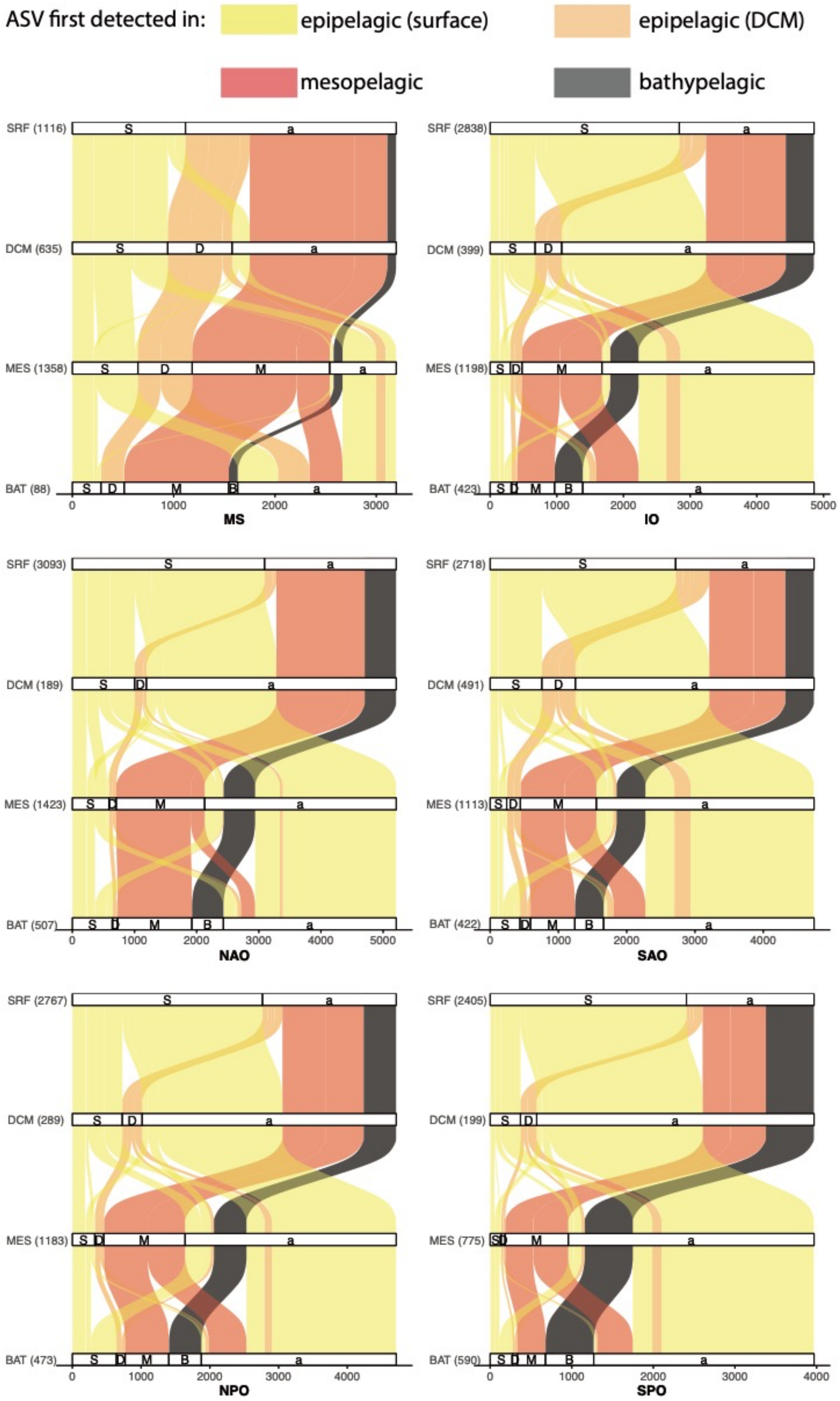

ASV first detected in:
epipelagic (surface)
epipelagic (DCM)
mesopelagic
bathypelagic
SRF (1116)
DCM (635)
MES (1358)
BAT (88)
MS
SRF (2838)
DCM (399)
MES (1198)
BAT (423)
IO
SRF (3093)
DCM (189)
MES (1423)
BAT (507)
NAO
SRF (2718)
DCM (491)
MES (1113)
BAT (422)
SAO
SRF (2767)
DCM (289)
MES (1183)
BAT (473)
NPO
SRF (2405)
DCM (199)
MES (775)
BAT (590)
SPO

Supplementary Figure 1: ASVs across depth layers. For each region, we color ASVs based on the layer they first appeared: Surface [SRF] (S, yellow), DCM (D, orange), Mesopelagic [MES] (M, red), and Bathypelagic [BAT] (B, black). Next to each layer, the number of ASVs that were first detected in that layer is indicated. Absent ASVs are grouped in box “a”. An ASV, only appearing in the bathypelagic, is assigned to box “a” in the above layers. That is, an ASV detected in the surface and present in the DCM but absent in lower layers appears in box (S) in the surface and DCM layer but in box “a” in the meso- and bathypelagic layer. An ASV cannot be assigned to two layers. Note that most ASVs in the bathypelagic zone have already been detected in upper layers because most ASVs are assigned to the boxes “S”, “D”, and “M” instead of “B”. See specific details in the GitHub/Zenodo repositories (https://doi.org/10.5281/zenodo.10230073) [section 06_Vertical Connectivity, Additional Tables]. Source data are provided in the GitHub/Zenodo repositories (section 06_Vertical Connectivity, https://doi.org/10.5281/zenodo.10230073; see Data Availability).

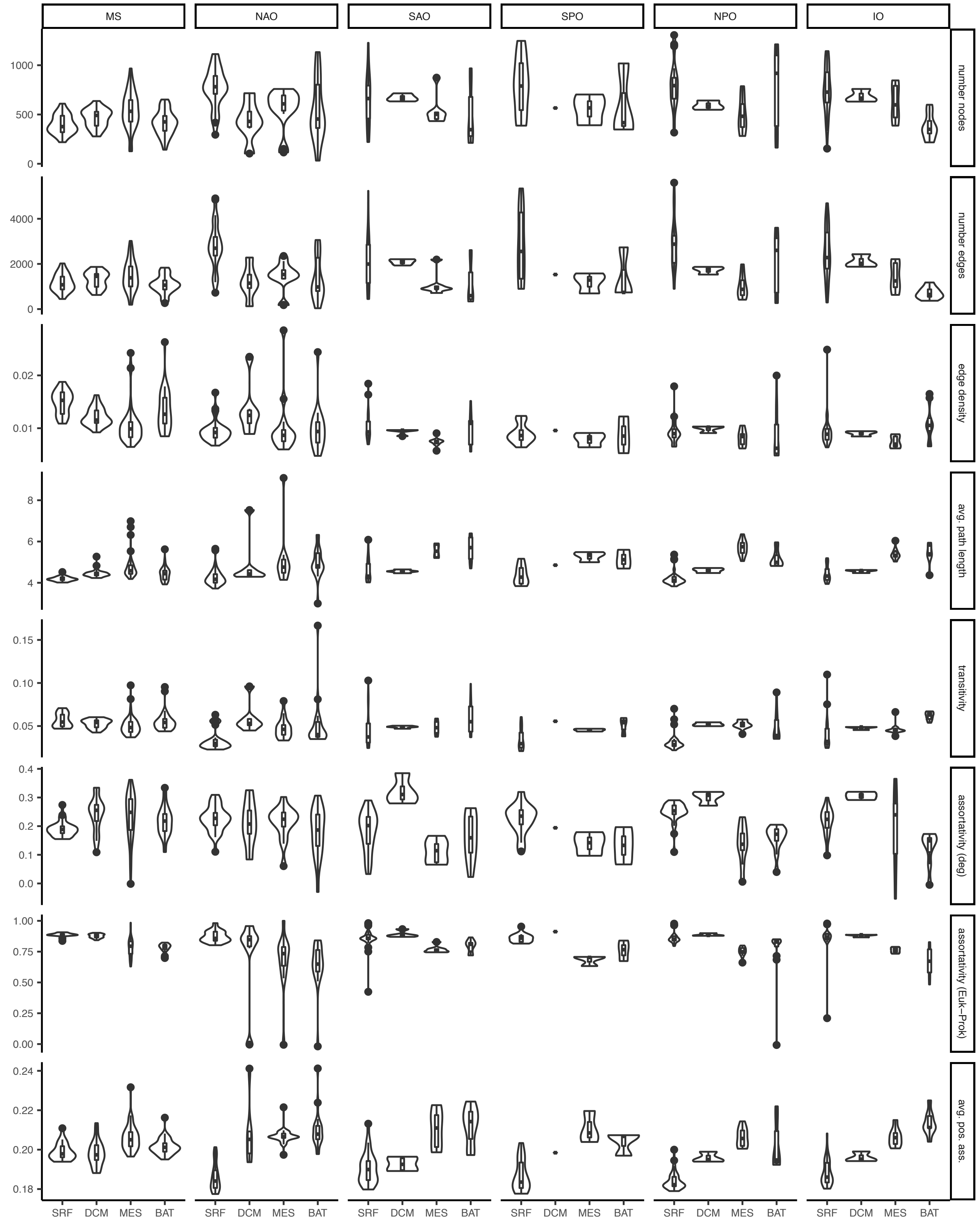

Supplementary Figure 2: Network metrics grouped by region and depth layer. Source data are provided in the GitHub/Zenodo repositories (section 03_GlobalNetworkMetrics, https://doi.org/10.5281/zenodo.10230073; see Data Availability).

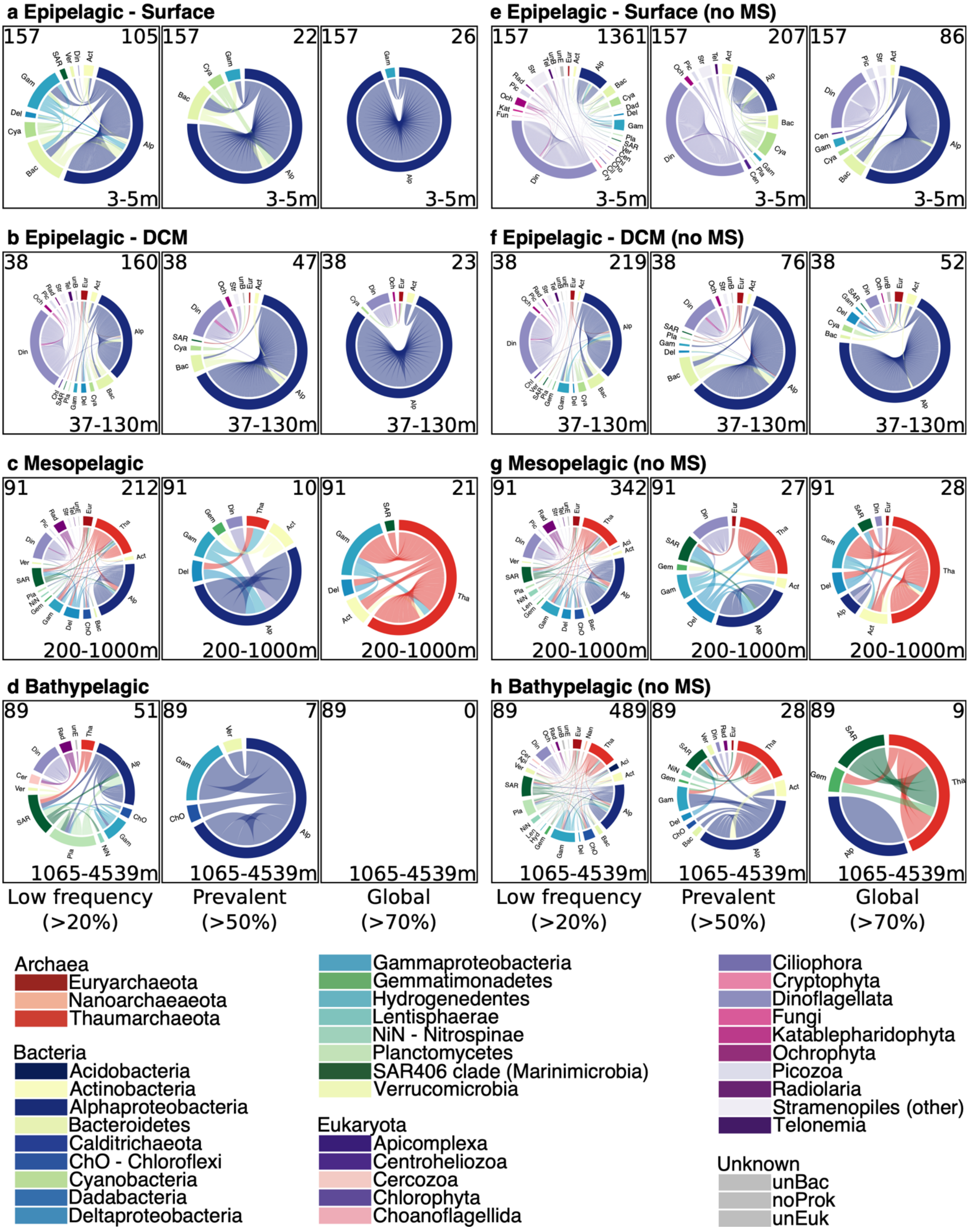

a Epipelagic - Surface
157
105
157
22
157
26
3-5m
3-5m
3-5m
e Epipelagic - Surface (no MS)
157
1361
157
207
157
86
3-5m
3-5m
3-5m
b Epipelagic - DCM
38
160
38
47
38
23
37-130m
37-130m
37-130m
f Epipelagic - DCM (no MS)
38
219
38
76
38
52
37-130m
37-130m
37-130m
c Mesopelagic
91
212
91
10
91
21
200-1000m
200-1000m
200-1000m
g Mesopelagic (no MS)
91
342
91
27
91
28
200-1000m
200-1000m
200-1000m
d Bathypelagic
89
51
89
7
89
0
1065-4539m
1065-4539m
1065-4539m
h Bathypelagic (no MS)
89
489
89
28
89
9
1065-4539m
1065-4539m
1065-4539m
Low frequency (>20%)
Prevalent (>50%)
Global (>70%)
Low frequency (>20%)
Prevalent (>50%)
Global (>70%)
Archaea
Euryarchaeota
Nanoarchaeaeota
Thaumarchaeota
Bacteria
Acidobacteria
Actinobacteria
Alphaproteobacteria
Bacteroidetes
Calditrichaeota
ChO - Chloroflexi
Cyanobacteria
Dadabacteria
Deltaproteobacteria
Gammaproteobacteria
Gemmatimonadetes
Hydrogenedentes
Lentisphaerae
NiN - Nitrospinae
Planctomycetes
SAR406 clade (Marinimicrobia)
Verrucomicrobia
Eukaryota
Apicomplexa
Centroheliozoa
Cercozoa
Chlorophyta
Choanoflagellida
Ciliophora
Cryptophyta
Dinoflagellata
Fungi
Katablepharidophyta
Ochrophyta
Picozoa
Radiolaria
Stramenopiles (other)
Telonemia
Unknown
unBac
noProk
unEuk

Supplementary Figure 3: Associations occurring in each region and depth layer and their taxonomy. If an association appears in more than 20% of subnetworks in each region, it is classified as low-frequency, >50% prevalent, and >70% global. The number of samples appear in the upper left corner, the number of edges in the upper right corner, and the depth range in the lower right corner (in meters [m] below the surface). We classified the associations considering all six regions **a - d** and considering the five ocean basins without the MS, **e - h**. Source data are provided in the GitHub/Zenodo repositories (section 05_ClassifyingAssociations, https://doi.org/10.5281/zenodo.10230073; see Data Availability).

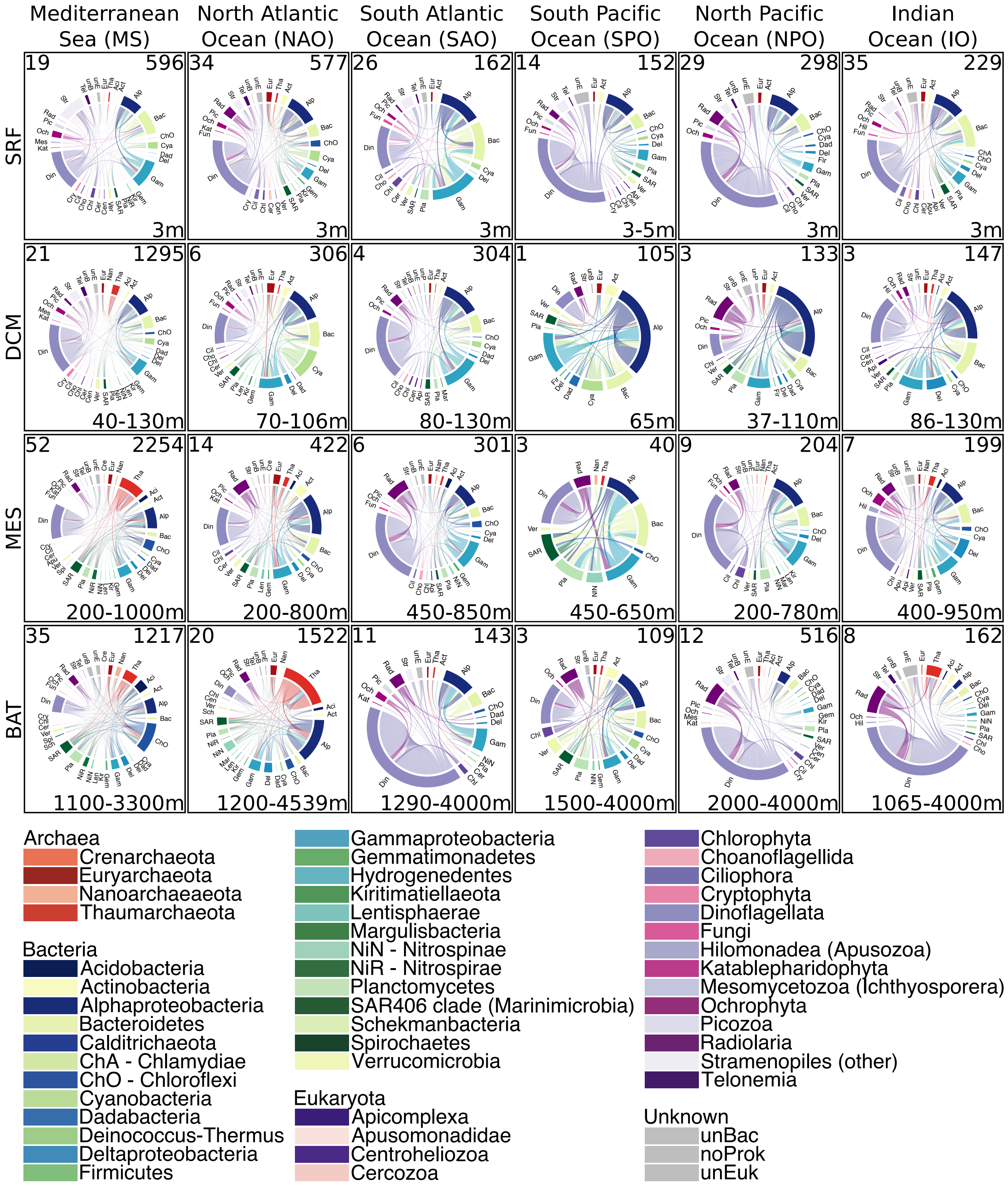

Supplementary Figure 4: Regional associations occurring in each region and depth layer and their taxonomy. Within a particular depth layer, if an association appears in at least one subnetwork (present) in one region and in no subnetwork (absent) in other regions, it is classified as regional. The four ocean layers (rows) are surface (SRF), DCM, mesopelagic (MES), and bathypelagic (BAT). The number of samples appear in the upper left corner, the number of edges in the upper right corner, and the depth range in the lower right corner (in meters [m] below the surface). Source data are provided

in the GitHub/Zenodo repositories (section 05_ClassifyingAssociations, https://doi.org/10.5281/zenodo.10230073; see Data Availability).

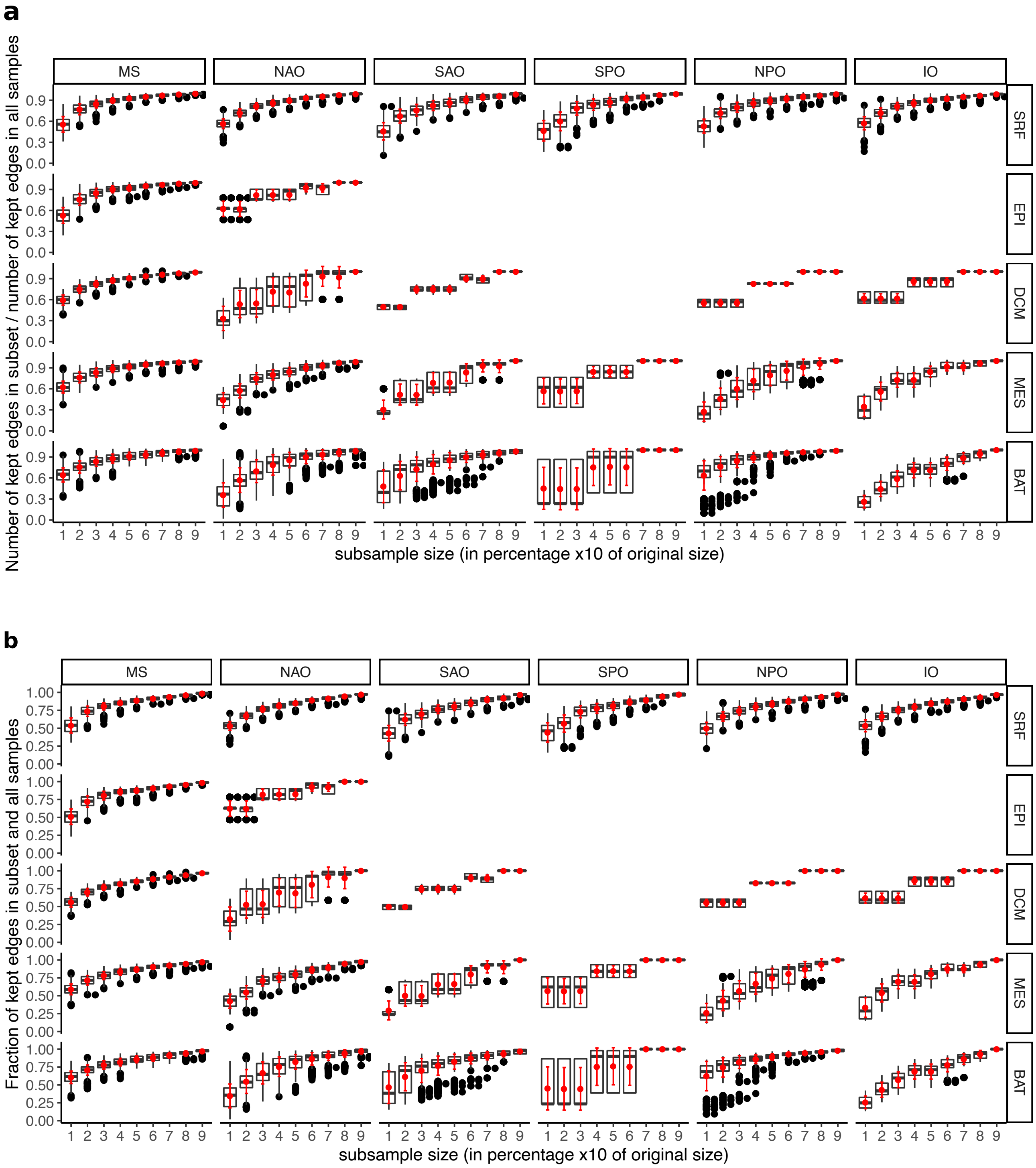

Supplementary Figure 5: Robustness check. Robustness of the third condition for generating sample-specific subnetworks for each region and depth with sufficient samples (the DCM layer from the SPO was removed because it contained only one sample). Within each region and depth, the samples were randomly subsampled, containing 10% to 90% of the original set using all samples. The y-axis shows the fraction of edges kept in the subsampled set compared to the original set. We

considered **a** only the number of kept edges and **b** which edges were kept. Source data are provided in the GitHub/Zenodo repositories (section 02_NetworkConstruction, https://doi.org/10.5281/zenodo.10230073; see Data Availability).

Supplementary Tables

Supplementary Table 1: Number of ASVs in the layer where they were first detected (from surface to bottom) for the different regions. The ASVs unique to specific depth layers are shown in brackets. Note that ASVs from the upper layers can be present in the lower layers, but not vice versa. For example, the 1358 ASVs first detected in the Mesopelagic of the Mediterranean Sea (MES) can be present in the Bathypelagic but not in the DCM. In turn, the ASVs that are unique to specific depth layers (in brackets) are only present in the specified layer. MS – Mediterranean Sea, IO – Indian Ocean, NAO – North Atlantic Ocean, SAO – South Atlantic Ocean, NPO – North Pacific Ocean, SPO – South Pacific Ocean.

	**MS**	**IO**	**NAO**	**SAO**	**NPO**	**SPO**
**Surface**	1116 (103)	2838 (2005)	3093 (1821)	2718 (1807)	2767 (1809)	2405 (1870)
**DCM**	635 (95)	399 (212)	189 (52)	491 (239)	289 (109)	199 (112)
**Mesopelagic**	1358 (326)	1198 (632)	1423 (227)	1113 (464)	1183 (547)	775 (431)
**Bathypelagic**	88 (88)	423 (423)	507 (507)	422 (422)	473 (473)	590 (590)

Supplementary Table 2: Fraction of microbial associations across depth layers. For each region and layer (rows), we determined the associations (in percentage %), classifying them based on their first appearance (columns): surface (SRF), DCM, mesopelagic, and bathypelagic. We indicated the fractions above 40% in grey. MS – Mediterranean Sea, NAO – North Atlantic Ocean, SAO – South Atlantic Ocean, SPO – South Pacific Ocean, NPO – North Pacific Ocean, IO – Indian Ocean.

Region	Layer	Surface	DCM	Mesopelagic	Bathypelagic
MS	SRF	100.00			
	DCM	45.14	54.86		
	Mesopelagic	10.35	18.42	71.24	
	Bathypelagic	2.73	5.12	69.71	22.44
NAO	SRF	100.00			
	DCM	68.30	31.70		
	Mesopelagic	11.64	6.59	81.77	
	Bathypelagic	11.62	1.35	43.49	43.54
SAO	SRF	100.00			
	DCM	45.08	54.92		
	Mesopelagic	6.15	8.50	85.35	
	Bathypelagic	12.22	6.30	26.97	54.61
SPO	SRF	100.00			
	DCM	50.07	49.93		
	Mesopelagic	6.44	2.66	90.90	
	Bathypelagic	9.81	3.32	14.15	72.71
NPO	SRF	100.00			
	DCM	54.23	45.77		
	Mesopelagic	8.33	6.06	85.61	
	Bathypelagic	17.46	5.34	19.92	57.28
IO	SRF	100.00			
	DCM	39.23	60.77		
	Mesopelagic	5.92	7.87	86.21	
	Bathypelagic	11.00	3.84	29.61	55.56

Supplementary Table 3: Subnetwork clusters. Clusters dominated, i.e., over 50%, by one layer or one region are indicated in grey. The last row shows unassigned subnetworks.

cluster ID	Dominated by	Size	Fraction of depth layers: Epipelagic SRF	Epipelagic EPI	Epipelagic DCM	Meso-pelagic	Bathy-pelagic	Number of regions (if no number if indicated, it is 1x): Epipelagic SRF	Epipelagic EPI	Epipelagic DCM	Meso- MES	Bathy- BAT
1	MS	5	20.00	20.00	20.00	20.00	20.00	SAO	MS	NAO	MS	MS
2	MS	10	10.00	-	20.00	20.00	50.00	MS	-	2xMS	2xMS	5xMS
3	MS	8	12.50	-	-	25.00	62.50	SRF	-	-	2xMS	5xMS
4	MS, MES	8	-	12.50	-	75.00	12	-	MS	-	6xMS	MS
5	MS, MES	12	16.67	-	-	66.67	16.67	IO, NAO	-	-	7xMS, NAO	2xNAO
6		8	12.50	25.00	12.50	25.00	25.00	IO	MS, NAO	NPO	MS, NAO	2xMS
7	BAT	15	13.33	-	-	26.67	60.00	IO, SPO	-	-	IO, MS, SAO, SPO	IO, MS, NAO, 2xNPO, 2xSAO, 2xSPO
8	DCM	10	10.00	-	90.00	-	-	NPO	-	5xMS, NPO, 3xSAO	-	-
9	DCM	11	36.36	-	63.64	-	-	2xNAO, NPO, SAO	-	3xIO, 2xMS, NPO, SAO	-	-
10		12	-	-	8.33	50.00	41.67	-	-	NAO	IO, MS, NAO, 2xNPO, SAO	IO, 2xNAO, NPO, SAO
11	MES	6	-	-	-	83.33	16.67	-	-	-	IO, MS, NPO, 2xSAO	IO
12	NAO, MES	6	16.67	-	-	83.33	-	NAO	-	-	2xMS, 3xNAO	-
13	SRF	11	54.55	9.09	-	27.27	9.09	IO, MS, NPO, 3xSAO	MS	-	2xMS, NAO	MS
14	BAT	16	12.50	6.25	6.25	6.25	68.75	MS, NAO	MS	MS	MS	5xNAO, 3xNPO, 2xSAO, SPO
15	SRF	8	100.00	-	-	-	-	3xIO, 4xNAO, NPO	-	-	-	-
16	MS, SRF	7	71.43	14.29	-	14.29	-	4xMS, NPO	MS	-	MS	-
17	MS	9	-	11.11	33.33	22.22	33.33	-	MS	MS, NAO, SPO	2xMS	3xMS
18	MS, BAT	8	12.50	25.00	-	-	62.50	IO	2xMS	-	-	3xMS, 2xNAO
19	SRF	7	85.72	14.29	-	-	-	2xIO, NAO, NPO, 2xSAO	MS	-	-	-
20	SRF	15	73.33	-	6.67	6.67	13.33	2xIO, 2xNAO, NPO, 5xSAO, SPO	-	MS	IO	IO, NPO
21		8	25.00	-	12.50	25.00	37.50	IO, SPO	-	MS	MS, SAO	IO, 2xNAO
22		17	23.53	-	5.88	35.29	35.29	3xSAO, SPO	-	MS	NAO, 2xNPO, SAO, 2xSPO	IO, MS, NAO, 3xSAO
23	SRF	8	75.00	12.50	-	12.50	-	IO, 2xMS, NAO, NPO, SPO	MS	-	MS	-
24	MS, MES	13	15.38	7.69	-	61.54	15.38	2xMS	MS	-	IO, 4xMS, 3xNAO	NAO, NPO
25		14	28.57	7.14	14.29	7.14	42.86	2xMS, 2xNAO	MS	2xMS	NAO	MS, 3xNPO, 2xSAO
26	SRF	7	85.72	14.29	-	-	-	2xIO-SRF, MS-EPI, 2xNAO-SRF, 2xNPO-SRF	2xIO-SRF, MS-EPI, 2xNAO-SRF, 2xNPO-SRF	-	-	-
27	SRF	11	100.00	-	-	-	-	2xIO, NAO, 4xNP, 4xSPO	-	-	-	-
28	MS	11	9.09	27.27	-	36.36	27.27	MS	3xNAO	-	4xMS	3xMS
29		12	50.00	-	16.67	16.67	16.67	IO, MS, 3xNAO, SAO	-	MS, NAO	2xMS	2xMS
30		6	50.00	-	16.67	16.67	16.67	IO, NAO, SPO	-	MS	NPO	IO-BAT
31	MS	28	25.00	10.71	7.14	35.71	21.43	4xIO, 2xMS, SAO	3xMS	2xMS	6xMS, 2xNAO, 2xNPO	IO, 2xMS, 3xNAO
32	SRF	6	100.00	-	-	-	-	IO, 2xNA, NPO, 2xSAO	-	-	-	-
33	SRF	6	100.00	-	-	-	-	NAO, 3xNPO, SAO, SPO	-	-	-	-
34	SRF	14	100.00	-	-	-	-	IO, 4xNAO, 5xNPO, 2xSAO, 2xSPO	-	-	-	-
35	SRF	13	69.23	7.69	-	-	23.08	4xIO, 3xNAO, SAO, SPO	MS	-	-	3xMS
36	SRF	7	100.00	-	-	-	-	3xIO, 3xNPO, SAO	-	-	-	-
-		24	41.67	-	12.50	29.17	16.67	2xIO, MS, 2xNAO, 3xNPO, 2xSAO	-	MS, 2xNAO	2xIO, 4xMS, NPO	MS, NAO, NPO, SAO

MS – Mediterranean Sea, NAO – North Atlantic Ocean, SAO – South Atlantic Ocean, SPO – South Pacific Ocean, NPO – North Pacific Ocean, IO – Indian Ocean, EPI – epipelagic layer, SRF – surface, DCM – Deep Chlorophyll Maximum, MES – mesopelagic layer, BAT – bathypelagic layer

Supplementary Table 4: Number of environmentally-driven edges detected by EnDED. We removed environmentally-driven edges (indirect) from the preliminary network, which contained 31966 edges. Only edges not environmentally driven by any environmental factor (not indirect) remained in the network.

Environmental factor	Number of samples	indirect	Not indirect
Fluorescence	394	4 (0.01%)	31962
NO_3	361	1563 (4.9%)	30403
PO_4	359	1357 (4.2%)	30609
Salinity	395	67 (0.2%)	31899
SiO_4	360	632 (2.0%)	31334
Temperature	395	622 (1.9%)	31344
All		2848 (8.9%)<br>= 1779 removed by 1<br>+ 751 removed by 2<br>+ 308 removed by 3<br>+ 10 removed by 4	29118 (91.1%)

Supplementary Table 5: Edge filtering. Number of edges within each region and depth layer before (J>0%) and after filtering edges with low Jaccard index measuring how often the association partners appeared together in the region and depth layer. The DCM layer in the South Pacific Ocean (SPO) contained only one subnetwork, which resulted in the edge prevalence being 100% for all edges. We selected the Jaccard index of J>20% to generate the sample-specific subnetworks.

Region	Layer	Samples	Depth (m)	J>0%	J>10%	J>20%	J>30%	J>40%	J>50%
MS	EPI - SRF	19	3	3710	3631	3263	2881	2375	1797
	EPI	18	12-50	4763	4682	4196	3731	3064	2189
	EPI - DCM	21	40-130	5545	5417	4736	4030	3062	2027
	MES	52	200-1000	8756	8403	7336	6179	4629	3088
	BAT	35	1100-3300	4497	4263	3694	3171	2506	1830
NAO	EPI - SRF	34	3	15862	15255	13478	11449	8487	5331
	EPI	4	50	3027	3027	3027	2778	2529	2091
	EPI - DCM	6	70-106	3865	3865	3738	3480	2973	2212
	MES	14	200-800	6325	6289	5689	5109	4169	2978
	BAT	20	1200-4539	7490	7419	6831	6206	5211	3857
SAO	EPI - SRF	26	3	13118	12768	11026	9269	6842	4353
	EPI - DCM	4	80-130	4199	4199	4199	3941	3443	2468
	MES	6	450-850	3937	3937	3740	3440	2687	1614
	BAT	11	1290-4000	4143	4130	3886	3605	3049	2254
NPO	EPI - SRF	29	3	14376	13778	11919	9907	7323	4736
	EPI - DCM	3	37-110	3100	3100	3100	3100	2568	1968
	MES	9	200-780	4197	4197	3781	3343	2583	1625
	BAT	12	2000-4000	5198	5185	4834	4510	4009	3372
SPO	EPI - SRF	14	3-5	12007	11927	10420	8990	6728	4480
	EPI - DCM	1	65	1530	1530	1530	1530	1530	1530
	MES	3	450-650	2066	2066	2066	2066	1756	1318
	BAT	3	1500-4000	3159	3159	3159	3159	2906	2128
IO	EPI - SRF	35	3	14307	13646	11736	9602	6912	4396
	EPI - DCM	3	86-130	3411	3411	3411	3411	2855	2310
	MES	7	400-950	4654	4654	4344	3961	3083	2082
	BAT	8	1065-4000	2928	2928	2790	2563	2101	1290

MS – Mediterranean Sea, NAO – North Atlantic Ocean, SAO – South Atlantic Ocean, SPO – South Pacific Ocean, NPO – North Pacific Ocean, IO – Indian Ocean, EPI – epipelagic layer, SRF – surface, DCM – Deep Chlorophyll Maximum, MES – mesopelagic layer, BAT – bathypelagic layer
