# Supplementary material for: Disentangling microbial networks across pelagic zones in the tropical and subtropical global ocean
Source: Nat Commun. 2024 Jan 2;15:126. doi: 10.1038/s41467-023-44550-y (PMC10762198; doi:10.1038/s41467-023-44550-y)
Supplement: Supplementary file 3 — Description of Additional Supplementary Files [file 41467_2023_44550_MOESM3_ESM.pdf]

Description of Additional Supplementary Files

Supplementary Data 1: Highly prevalent (>70%) associations per region and depth layer. For each association between two taxa (first and second columns), we list the frequency (third column) and percentage (fourth column) concerning region (fifth column) and depth layer (sixth column).

Supplementary Data 2: Highly prevalent (>70%) regional associations. For each association between two ASVs (first and second columns), we list: region (third column), depth layer (fourth column), the prevalence in that region and depth layer (fifth column), type: eukaryotic (Euk_Euk), prokaryotic (Prok_Prok), and the association between domains (Euk_Prok) (sixth column), and the phyla (seventh and eight columns).

Supplementary Data 3: Associations appearing in all layers in at least one region. For each association between two ASVs (first and second columns), we list: the classification in each layer (3-6 columns), overall prevalence (8. column), prevalence in each region, and depth layer (9- 34. columns), the number of regions in which the association appeared in all layers (AllLayers, 35. column), the number of layers an association appears in a region (36-41. columns), type: eukaryotic (Euk_Euk), prokaryotic (Prok_Prok), and the association between domains (Euk_Prok) (42. column), and the phyla (43-44. column).
