# Supplementary material for: Disentangling microbial networks across pelagic zones in the tropical and subtropical global ocean
Source: Nat Commun. 2024 Jan 2;15:126. doi: 10.1038/s41467-023-44550-y (PMC10762198; doi:10.1038/s41467-023-44550-y)
Supplement: Supplementary file 7 — Reporting Summary [file 41467_2023_44550_MOESM7_ESM.pdf]

nature portfolio

Corresponding author(s): Ina Deutschmann
Ramiro Logares

Last updated by author(s): Nov 23, 2023

Reporting Summary

Nature Portfolio wishes to improve the reproducibility of the work that we publish. This form provides structure for consistency and transparency in reporting. For further information on Nature Portfolio policies, see our Editorial Policies and the Editorial Policy Checklist.

Statistics

For all statistical analyses, confirm that the following items are present in the figure legend, table legend, main text, or Methods section.

n/a	Confirmed	
[ ]	[x]	The exact sample size (*n*) for each experimental group/condition, given as a discrete number and unit of measurement
[ ]	[x]	A statement on whether measurements were taken from distinct samples or whether the same sample was measured repeatedly
[x]	[ ]	The statistical test(s) used AND whether they are one- or two-sided<br>*Only common tests should be described solely by name; describe more complex techniques in the Methods section.*
[x]	[ ]	A description of all covariates tested
[ ]	[x]	A description of any assumptions or corrections, such as tests of normality and adjustment for multiple comparisons
[ ]	[x]	A full description of the statistical parameters including central tendency (e.g. means) or other basic estimates (e.g. regression coefficient) AND variation (e.g. standard deviation) or associated estimates of uncertainty (e.g. confidence intervals)
[x]	[ ]	For null hypothesis testing, the test statistic (e.g. *F*, *t*, *r*) with confidence intervals, effect sizes, degrees of freedom and *P* value noted<br>*Give P values as exact values whenever suitable.*
[x]	[ ]	For Bayesian analysis, information on the choice of priors and Markov chain Monte Carlo settings
[x]	[ ]	For hierarchical and complex designs, identification of the appropriate level for tests and full reporting of outcomes
[x]	[ ]	Estimates of effect sizes (e.g. Cohen's *d*, Pearson's *r*), indicating how they were calculated

Our web collection on statistics for biologists contains articles on many of the points above.

Software and code

Policy information about availability of computer code

Data collection	No special software was used.
Data analysis	Used software: DADA2 v1.20, Flashweave v0.18.0, EnDED v1.0.1, Julia v1.5.3, R v4.0.x, iGraph v1.2.6 (R-package), Gephi v0.9.2, Python v3x, UMAP v0.5.2 (Python-package), hdbscan v0.8.27 (Python-package). All the code used in the analyses has been deposited in GitHub (https://github.com/InaMariaDeutschmann/GlobalNetworkMalaspinaHotmix) and Zenodo (https://doi.org/10.5281/zenodo.10230073).

For manuscripts utilizing custom algorithms or software that are central to the research but not yet described in published literature, software must be made available to editors and reviewers. We strongly encourage code deposition in a community repository (e.g. GitHub). See the Nature Portfolio guidelines for submitting code & software for further information.

Data

Policy information about availability of data

All manuscripts must include a data availability statement. This statement should provide the following information, where applicable:
- Accession codes, unique identifiers, or web links for publicly available datasets
- A description of any restrictions on data availability
- For clinical datasets or third party data, please ensure that the statement adheres to our policy

DNA sequence data is publicly available at the European Nucleotide Archive (https://www.ebi.ac.uk/ena; see details in Table 2). The accession numbers for the different datasets are: MalaSurf (PRJEB23913, PRJEB25224), MalaVP (PRJEB23771, PRJEB45015), MalaDeep (PRJEB45011, PRJEB45014), Hotmix (PRJEB44683, PRJEB44474). The following databases have been used: SILVA v132 (https://www.arb-silva.de/no_cache/download/archive/release_132/) , PR2 v4.11.1 (https://

github.com/pr2database/pr2database/releases), and the World Ocean Database 2013 (doi:10.7289/V5NZ85MT).

Research involving human participants, their data, or biological material

Policy information about studies with human participants or human data. See also policy information about sex, gender (identity/presentation), and sexual orientation and race, ethnicity and racism.

Reporting on sex and gender	This information has not been collected (not relevant for the study)
Reporting on race, ethnicity, or other socially relevant groupings	This information has not been collected (not relevant for the study)
Population characteristics	This information has not been collected (not relevant for the study)
Recruitment	This information has not been collected (not relevant for the study)
Ethics oversight	This information has not been collected (not relevant for the study)

Note that full information on the approval of the study protocol must also be provided in the manuscript.

Field-specific reporting

Please select the one below that is the best fit for your research. If you are not sure, read the appropriate sections before making your selection.

☐ Life sciences ☐ Behavioural & social sciences ☒ Ecological, evolutionary & environmental sciences

For a reference copy of the document with all sections, see nature.com/documents/nr-reporting-summary-flat.pdf

Ecological, evolutionary & environmental sciences study design

All studies must disclose on these points even when the disclosure is negative.

Study description	We investigated the potential ecological interactions among marine microorganisms, including archaea, bacteria, and picoeukaryotes, in different depths and geographical regions of the tropical and subtropical global ocean and the Mediterranean Sea. In total, we analyzed 397 marine plankton samples from different ocean depths, using 16S and 18S rRNA metabarcoding. Samples were obtained in two oceanographic surveys from the expeditions Malaspina 2010 and Hotmix. We discovered that potential microbial interactions change with depth and geographical scale, displaying highly heterogeneous distributions . We found that only a few potential interactions were global, while 11-36% were regional within specific depths. We also found that most surface water associations do not persist in deeper ocean layers, despite microbial vertical connectivity.
Research sample	We sampled microbial plankton at different oceanic depths aiming to determine community composition. Sampling stations were chosen to cover the tropical and subtropical global ocean, and the Mediterranean Sea, targeting different depth layers (from the surface up to ca. 4000m depth). DNA was extracted from the samples and used for sequencing the 18S and 16S rRNA markers. Taxonomic analyses of the produced sequences were used to determine the microbial community composition in each plankton sample.
Sampling strategy	Sampling stations were selected during the global oceanographic campaign Malaspina 2010 and the regional campaign Hotmix, focusing in the Mediterranean Sea. Stations were separated by 200-700 km, and the sampling approach aimed to recover samples from different depths, covering the entire water column (from surface to the deep ocean). We focused on the smallest ocean plankton, the pico-plankton, which considers organisms with cells sizes between 0.2-3 µm. We focused on the pico-plankton as they are key for marine ecosystem function. A few samples targeted the 0.2-0.8 µm and 0.8-20 µm size fractions. Given the cell sizes of prokaryotes versus microeukaryotes, we used the smallest size-fraction (0.2-0.8 µm) for prokaryotes and the larger one (0.8-20 µm) for microbial eukaryotes. Size fractionation was done by sequential filtering of the water through filters with different pore size.
Data collection	Microbial plankton samples were obtained by filtering water and collecting the biomass onto filters. Sequential filtering through filters with different pore size was used to target the pico-plankton size-fraction (considering organisms with cell sizes in between 0.2-3 µm). Filtering was done on-board the R/V Hespérides and the R/V Sarmiento de Gamboa by the scientific crews that were part of the expeditions. Community DNA extraction was done at the ICM CSIC by laboratory technicians. Community DNA was then used for sequencing the 16S and 18S rRNA markers at specialized DNA-sequencing centers.
Timing and spatial scale	Samples originated from two oceanographic expeditions: Malaspina-2010 and Hotmix . The Malaspina expedition aimed at sampling the tropical and subtropical global ocean, and took place onboard the R/V Hespérides. Most ocean basins were sampled between December 2010 and July 2011. Sampling was organized so that most regions were sampled at similar meteorological seasons. The geographic scope of the Malaspina expedition was the tropical and subtropical global ocean, which was determined during the planning of this campaign, considering the available ship time and budget. For the Hotmix expedition, sampling took place onboard the R/V Sarmiento de Gamboa between 27th April and 29th May 2014 and represented a quasi-synoptic transect across the Mediterranean Sea and the adjacent North-East of the North Atlantic Ocean. The Hotmix expedition was designed to cover the entire Mediterranean Sea, thus having a regional geographic scope form the beginning. In addition, the Hotmix campaign occurred during the same season, thus reducing the effects of seasonality on the measured marine microbial communities.
Data exclusions	No data were excluded from the analyses

Reproducibility	Attempts at replication were successful; we have run analyses multiple times and arrived to the same results. The code for data analysis to reproduce our results, including commands to run FlashWeave and EnDED (environmentally-driven-edge-detection), is publicly available at GitHub (https://github.com/InaMariaDeutschmann/GlobalNetworkMalaspinaHotmix) and Zenodo (https://doi.org/10.5281/zenodo.10230073). OTU tables and results from specific analyses are provided in GitHub (https://github.com/InaMariaDeutschmann/GlobalNetworkMalaspinaHotmix) and Zenodo (https://doi.org/10.5281/zenodo.10230073). DNA sequence data is publicly available at the European Nucleotide Archive. The accession numbers for the different datasets are: MalaSurf (PRJEB23913, PRJEB25224), MalaVP (PRJEB23771, PRJEB45015), MalaDeep (PRJEB45011, PRJEB45014), Hotmix (PRJEB44683, PRJEB44474).
Randomization	Our sampling approach consisted in a large-scale field survey carried out in oceanographic ships. Here, samples are taken along the ship's trajectory. Randomization was not relevant to our field survey, as we did not need to allocate samples to groups. Note that in most oceanographic campaigns, the selection of stations is not done randomly. Instead, stations are carefully selected based on physicochemical or biological conditions. Randomization was carried out in specific bioinformatics analyses, such as when testing the third condition during network construction (see Methods in the main text of the manuscript). There, for example, we tested robustness (of the third condition in network construction) by randomly drawing a subset of samples from each oceanic region and depth combination (this is described in Methods in the main text).
Blinding	Our sampling approach consisted in a large-scale oceanographic survey, therefore blinding was not necessary, as our work is not experimental.

Did the study involve field work? ☒ Yes ☐ No

Field work, collection and transport

Field conditions	Samples originated from two oceanographic cruises, Malaspina-2010 and Hotmix . The former took place onboard the R/V Hespérides, and most ocean basins were sampled between December 2010 and July 2011. For the Hotmix expedition, sampling took place onboard the R/V Sarmiento de Gamboa between 27th April and 29th May 2014 and represented a quasi-synoptic transect across the Mediterranean Sea and the adjacent North-East of the North Atlantic Ocean. Plankton samples were taken at different depths zones: the epipelagic (0 to 200 meters), mesopelagic (200 to 1000 meters), and bathypelagic (1000 to 4000 meters). The field conditions at the sampling depth changed depending on the water mass that was sampled. In the global ocean, water temperature ranged between 1-4ºC in the bathypelagic zone, between 4-15ºC in the mesopelagic zone, and between 12-30ºC in the epipelagic zone. In turn, in the Mediterranean Sea, temperature was about 14ºC in the bathypelagic zone and above 14ºC in the meso- and epipelagic zones. Additional environmental variables measured at the sampling depths are show in Junger et al. 2023 (DOI:10.1126/sciadv.adg9763). Weather conditions during sampling at the surface were normally mild with no rain, as this is required to deploy the sampling instruments from the ship.
Location	We have sampled tropical and subtropical areas of five ocean basins: North and South Atlantic Ocean, North and South Pacific Ocean, and Indian Ocean. We have also sampled the Mediterranean Sea (Western and Eastern basins). Water samples were taken at different depth zones: the epipelagic zone (0 to 200 meters), mesopelagic zone (200 to 1000 meters), and bathypelagic zone (1000 to 4000 meters). More details on the sampling locations are shown in Figure 1.
Access & import/export	For the execution of the research presented in this study, efforts were made to ensure full compliance with local, national, and international laws and regulations pertinent to marine research as part of the large scale oceanographic campaigns Malaspina-2010 and Hotmix. Special permits were not needed for sampling marine microbes, as the field sampling occurred before the Nagoya protocol entered into force on October 2014. Nevertheless, the collected samples comply with the the Nagoya protocol as they are strictly used for biodiversity studies.
Disturbance	The study did not cause any disturbance.

Reporting for specific materials, systems and methods

We require information from authors about some types of materials, experimental systems and methods used in many studies. Here, indicate whether each material, system or method listed is relevant to your study. If you are not sure if a list item applies to your research, read the appropriate section before selecting a response.

Materials & experimental systems

n/a	Involved in the study	
☒	☐	Antibodies
☒	☐	Eukaryotic cell lines
☒	☐	Palaeontology and archaeology
☐	☒	Animals and other organisms
☒	☐	Clinical data
☒	☐	Dual use research of concern
☒	☐	Plants

Methods

n/a	Involved in the study	
☒	☐	ChIP-seq
☒	☐	Flow cytometry
☒	☐	MRI-based neuroimaging

Animals and other research organisms

Policy information about studies involving animals; ARRIVE guidelines recommended for reporting animal research, and Sex and Gender in Research

Laboratory animals	The study did not involve laboratory animals
Wild animals	The study did not involve wild animals
Reporting on sex	Not relevant (we investigate marine microbes)
Field-collected samples	Marine microbial cells were collected onto filters, which were stored onboard the oceanographic ships.
Ethics oversight	Samples comply with the the Nagoya protocol as they are use for biodiversity analysis.

Note that full information on the approval of the study protocol must also be provided in the manuscript.
